# Molecular epidemiology of hepatitis C infections in Ningxia, China: genotype, phylogeny and mutation analysis

**DOI:** 10.1186/s12985-016-0635-y

**Published:** 2016-10-18

**Authors:** Zhonglan Wu, Lijia Cui, Weiming Zhao, Dongzhi Yang, Hui Chen, Ruiqing Wang, Xuemin Wang, Linqi Zhang, Tianhua He

**Affiliations:** 1 Ningxia Center for Disease Control and Prevention, Ningxia, 750001 China; 2 Tsinghua University School of Medicine, Beijing, 100084 China; 3 Ningxia Medical University School of Public Health and Management, Ningxia, 750001 China; 4 Wuzhong Center of Disease Control and Prevention, Ningxia, 751100 China; 5 Comprehensive AIDS Research Center, and Collaborative Innovation Center for Diagnosis and Treatment of Infectious Diseases, Tsinghua University School of Medicine, Beijing, 100084 China

**Keywords:** Hepatitis C virus, Prevalence, Genotype, Phylogenetic, Genetic diversity

## Abstract

**Background:**

Current prevalence and genotype distribution of hepatitis C virus (HCV) infection remain unknown in Ningxia, northwest China.

**Methods:**

From June to December 2013, 13,022 individuals were screened in Ningxia HIV/AIDS Sentinel Surveillance System, with their demographic features collected and serum samples tested for HCV antibody. Sero-positive drug users were further subjected to sequencing of NS5B and Core regions of HCV.

**Results:**

The anti-HCV prevalence was 0.34 % among individuals without history of drug use, while it was 15.80 % among drug users. Of 79 NS5B sequences amplified from drug users, 64 (81.0 %) were male and 51 (64.0 %) were injection drug users (IDUs). Subtype 3a (40.5 %) and 1b (25.3 %) were the most predominant subtypes, followed in frequency by 3b (10.1 %) and 2a (7.6 %). Subtype distribution has no significant difference between injection and non-injection drug users. Based on phylogeographic analysis, HCV strains in Ningxia IDUs were mainly originated from two sites, Yunnan province (in southwest China bordering Myanmar, also known as Burma) and Xinjiang Autonomous Region (in northwest China on the border of Central Asia), which are the two major drug trafficking originates in China. Previously reported drug-resistance mutations were also scanned in this treatment-naïve population. Amino acid substitutions (C316N) associated with direct anti-viral agents (DAA) resistance were identified in the NS5B region in seven samples.

**Conclusion:**

This study is the first to reveal the existence of multiple genotypes of HCV in Ningxia, an inland province in northwest China, suggesting the rapid spreading of the virus.

**Electronic supplementary material:**

The online version of this article (doi:10.1186/s12985-016-0635-y) contains supplementary material, which is available to authorized users.

## Background

Hepatitis C is the second leading cause of chronic hepatitis, liver cirrhosis and hepatocellular carcinoma (HCC) in China. The seroprevalence of hepatitis C infection in China is about 0.43 % in general population, corresponding to 5.6 million people [1]. However, it has been estimated that the real seroprevalence of HCV may be close to 10 million in China [2]. In recent years, although the overall hepatitis C virus (HCV) prevalence is decreasing due to mandatory HCV antibody screening prior to blood transfusion [3–5], the prevalence among populations with high-risk behaviors remain high, especially among injection drug users (IDUs) [6, 7]. Studies have shown that the prevalence of hepatitis C among IDUs is 70 % in China [6, 8], compared to that of 60 % in the United States [9].

**Fig. 1:**
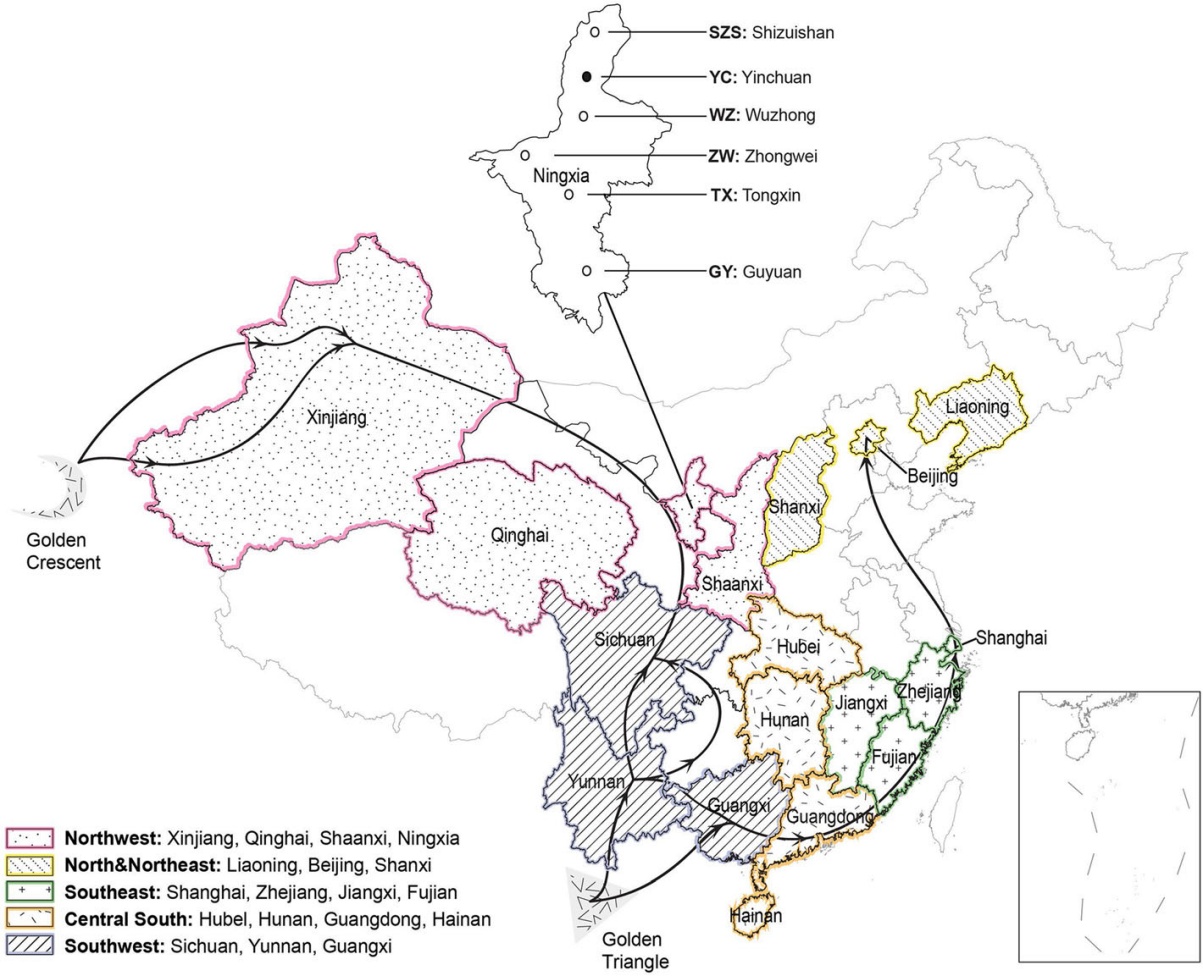
The map showing sampling spots in Ningxia and 17 provinces or municipalities in China, from which HCV reference sequences were included in the phylogenetic analysis. The 17 provinces and municipalities were divided into five regions and colored accordingly: the northwest (Xinjiang, Qinghai, Shaanxi), north & northeast (Liaoning, Beijing, Shanxi), southeast (Shanghai, Zhejiang, Jiangxi, Fujian), central south (Hubei, Hunan, Guangdong, Hainan), and southwest (Sichuan, Yunnan, Guangxi). Ningxia is located in northwest China, with six cities Shizuishan (SZS), Yinchuan (YC), Wuzhong (WZ), Zhongwei (ZW), Tongxin (TX), and Guyuan (GY) scattered from north to south where participants were recruited. Major drug trafficking routes were shown by grey arrows referring to Sullivan et al. 2007, which originated from Yunnan (bordering Myanmar and Laos) and Xinjiang (bordering Afghanistan and Pakistan), respectively [39]

HCV, a member of the family *Flaviviridae*, is a positive single-stranded RNA virus. HCV is mainly divided into 7 genotypes and more than 80 subtypes. HCV genotype 1, 2, and 3 are commonly distributed around the world [10]. Several nationwide studies have shown that genotype 1, 2, 3, 6 were the most prevalent genotypes in China [11, 12]. The current genetic diversity of HCV infection remains unclear in Ningxia Hui Autonomous Region, which is known for its ethnic diversity with about 64.58 % Han, 35.42 % Hui (Muslims) and less than 1 % other minorities (Fig. 1) [13]. Han is the dominant ethnic group in China with 90 % of the population, while most of the Hui ethnic minority inhabit in Ningxia, is descended from the Arabic and Persian merchants who came to China during the 7th century [14].

As HIV and HCV share the same routes of transmission, we recruited patients from Ningxia HIV/AIDS Sentinel Surveillance System, part of the nationwide disease management program in China launched in 1995 [15, 16]. The HIV/AIDS Sentinel Surveillance System is under the direction of the Ministry of Health (MOH) and the Chinese Center of Disease Control and Prevention (CDC), with nine key affecting populations (KAPs) were screened for HIV infections and related behaviors (Additional file 1: Supplementary Information 1). HCV testing was added and routinely performed since 2009 [17]. Blood samples, collected from KAPs registered from June to December 2013, were subjected to HCV tests, gene sequencing of NS5B, Core regions and further analysis.

HCV genotyping and phylogenetic analysis provide opportunity to reveal the recent HCV epidemic in Ningxia and the possible transmission networks in China. Such epidemiological and molecular information can also inform the efficacy of current regimens with Peginterferion and Ribavirin, and the upcoming highly effective direct anti-viral agents (DAAs) as well. Besides, few drug-resistance mutations have been reported in China, where no DAAs have not been approved yet. Therefore, pre-treatment testing for individuals may benefit in regimen choosing more effective regimens in the new DAA era.

## Methods

### Samples

Serum samples were collected from Ningxia HIV/AIDS Sentinel Surveillance System, where nine groups of key affecting populations (KAPs) were tested for HCV routinely, including 1) drug users (IDU and non-injection drug users), 2) blood donors, 3) female sex workers (FSW), 4) men who have sex with men (MSM), 5) sexual transmitted disease (STD) outpatients, 6) long-distance truck drivers, 7) migratory populations, 8) pregnant women and new mothers, and 9) young students, for HIV, HCV and syphilis screening (Additional file 1: Supplementary Information 1). In this study, only HCV-antibody positive individuals were further informed for detailed characteristics including age, ethnics, occupation, education and drug use history, using custom questionnaires.

### HCV antibody and RNA testing, and RNA sequencing

HCV antibody testing was conducted using enzyme-linked immunosorbant assay (ELISA) with HCV-IgG ELISA kit (Wantai, Beijing, China), and positive results were repeated twice for accuracy. HCV RNA was quantified by real-time PCR, using Roche light cycle 2.0 Diagnostic Kit for hepatitis C virus RNA (PCR-Fluorescence Probing) (Da’an, Shenzhen, China). HCV RNA positive was defined as RNA copies over 0.5 × 10^3^ IU/ml. Samples with enough leftovers were further sent for nested PCR and sequencing. Nested PCR was performed as follows: the first round RT-PCR was performed using One-Step RT-PCR kit (TransGene Biotech, Beijing, China) using NS5B and Core specific external primers with the following protocol: 45 °C 30 min, 94 °C 2 min, (94 °C 30s, 50 °C 30s, 72 °C 90s) × 35 cycles, and 72 °C 10 min; the second round PCR was performed using Promega Taq PCR Mastermix kit (Promega Company, USA), using 5ul product from the first round as the template, and NS5B and Core specific internal primers (Additional file 1: Supplementary Information 3 and Table S2), with the following protocol: (94 °C 2 min, 58 °C 30s, 72 °C 90s) × 30 cycles, and 72 °C 10 min. The final PCR product was tested in agar gel electrophoresis and sent for Sanger sequencing.

### HCV genotyping and mutation analysis

**Fig. 2:**
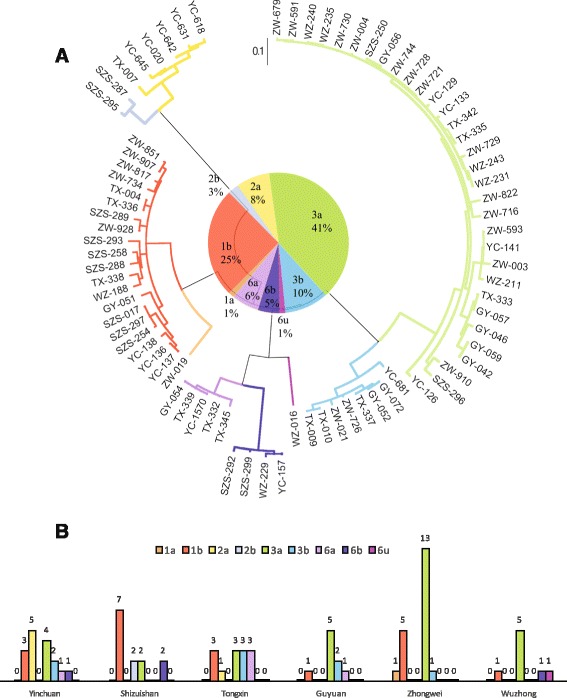
Subtype distribution of HCV based on the NS5B sequences from 79 subjects. **a** The circular phylogenetic tree was generated using the software MEGA 6.0, with maximum-likelihood (ML) method and GTR + I + Γ model with 500 bootstrap replications. HCV sequences were named by “city of serum collection” and “ID number”, where YC, SZS, TX, GY, ZW, and WZ standing for six cities in Ningxia, that is, Yinchuan, Shizuishan, Tongxin, Guyuan, Zhongwei, and Wuzhong, respectively. A pie chart showing the subtype distribution was combined with the tree topology. **b** A bar chart showing the subtype distribution by cities in Ningxia

Four hundred fifteen NS5B reference sequences of subtype 1a, 1b, 2a, 2b, 3a, 3b, 6a, 6b, 6n, 6u, 6v were downloaded from GenBank, of which accession numbers were KF585503 - KF585907, GQ206087, EU256071, AB661379, JQ745651, KC844048, EU408332, EU408331, EU408330, D84262, D37855. We performed sequence comparison using the software MEGA 6.0, with the method of Clustal W1.8, and constructed phylogenetic trees using maximum-likelihood (ML) method and GTR+I+Γ model [17–19]. The robustness of reconstructed phylogenies was evaluated by bootstrapping (500 bootstrap replications) to determine genotypes and calculate genetic distances between sequences. The tree topology based on NS5B and Core regions were displayed as a circular form combined with a pie chart of genotype distribution (Fig. 2a, Additional file 1: Figure S1). Of all NS5B sequence alignments, we scanned for previously published DAA-resistant mutations on NS5B.

### Phylogeographic analysis

Bayesian coalescent analysis was performed in BEAST software (v1.8.3). The GTR+I+gamma substitution model, uncorrected exponential clock model and Bayesian skygrid coalescent model were selected [20], and 5.0*10^−4^ substitution/site*year was set as the prior rate [21]. The XML file was generated by BEUTi program and ran in BEAST software, with MCMC run for 300,000,000 states and sampled every 10,000 states. The consensus tree was generated in TreeAnnotator program and Tracer program (v1.6.0), and deciphered in FigTree program (v1.4.2).

### Statistical analysis

We used R 3.0.2 for statistical analysis [22]. HCV subtype distribution was analyzed by Fisher’s exact test, and *p*-value less than 0.05 were considered as statistically significant. Kappa values were calculated to estimate the agreement of NS5B and Core genotyping methods (Additional file 1: Supplementary Information 2, and Table S1) [23, 24].

## Results

### Epidemiology

Among 13,022 individuals recruited from Ningxia HIV/AIDS Sentinel Surveillance System screened for anti-HCV antibody, 2443 were drug users. The overall seroprevalence was 0.34 % among people with no history of drug use, but as high as 5.8 % among drug users. 158 anti-HCV positive specimens were further tested for HCV RNA with 132 being RNA positive. The estimated HCV-RNA positive prevalence was 13.7 % for drug users (Table 1). The prevalence of HCV was significantly higher in IDUs, compared to non-injection drug users.

**Table 1:** HCV seroprevalence and RNA positive prevalence of the screened population by risk behaviors

Population by risk factors	Samples tested antibody	Samples antibody positive	Sero-prevalence	Samples tested RNA	HCV RNA+	Prevalence of HCV RNA+	Sequenced for NS5B region
Total	13,022	422	3.24 %	158	132	2.71 %	86
Drug users	2443	386	15.80 %	134	116	13.68 %	79
Injection drug user	601	284	47.25 %	82	73	42.07 %	51
Non-injection drug user	1842	102	5.54 %	52	43	4.58 %	28
Not Drug users	10,579	36	0.34 %	24	16	0.23 %	7
Blood donors	2099	3	0.14 %	0	0	-	0
Sex workers	2737	17	0.62 %	17	11	0.40 %	5
MSMs	400	0	0.00 %	0	0	-	0
Patients in STD clinics	2031	7	0.34 %	7	5	0.25 %	2
Long-distance truck drivers	100	2	2.00 %	0	0	-	0
Immigrants	400	1	0.25 %	0	0	-	0
Pregnant women	2012	2	0.10 %	0	0	-	0
College students	800	4	0.50 %	0	0	-	0

Nine groups from high- to low-risk populations were screened in Ningxia HIV/AIDS Sentinel Surveillance System, including 1) drug users (injection drug users and non-injection drug users), 2) blood donors, 3) female sex workers (FSW), 4) men who have sex with men (MSM), 5) sexual transmitted disease (STD) outpatients, 6) long-distance truck drivers, 7) migratory populations, 8) pregnant women, and 9) young students. The detailed criteria of the population were in Supplementary information 1. Prevalence of HCV antibody (seroprevalence) and HCV RNA was higher among injection drug users than that among non-injection drug users

### Demographic features

Among 79 drug users with a complete set of lab tests and genotype determined, 81.0 % of currently HCV-infected patients were male, 49.4 % were young adults (younger than 40 years old), 87.3 % had middle school or less education, and 69.6 % were unemployed. All drug users have a history of drug use longer than one year, and 64.6 % of them were IDUs. Nine patients (11.4 %), all IDUs, were also HIV co-infected. Detailed demographic characteristics and drug use information were summarized in Table 2.

**Table 2:** Genotype distribution of 79 drug users by demographic features

Genotype		All	1a	1b	2a	2b	3a	3b	6a	6b	6u	*p*-value^a^
Overall		79	1	20	6	2	32	8	5	4	1	
Sex												0.589
	Male	64	1	17	6	1	24	6	5	3	1	
	Female	15	0	3	0	1	8	2	0	1	0	
Age												**0.013**
	<30	14	1	4	2	0	5	1	1	0	0	0.843
	31–40	25	0	1	1	0	15	3	2	3	0	**0.010**
	41–50	36	0	15	2	2	11	3	1	1	1	**0.029**
	≥50	4	0	0	1	0	1	1	1	0	0	0.117
Marriage												**0.003**
	Unmarried	32	0	5	2	1	19	2	2	1	0	0.111
	Married	16	1	3	4	0	3	4	0	1	0	**0.004**
	Divorced or Widow	31	0	12	0	1	10	2	3	2	1	**0.035**
Ethnics												0.163
	Han	40	0	12	2	1	18	1	3	2	1	0.153
	Hui	31	0	5	3	1	11	7	2	2	0	**0.033**
	Others	8	1	3	1	0	3	0	0	0	0	0.721
Education												0.175
	Elementary school	35	1	10	5	0	9	3	4	3	0	**0.035**
	Middle school	34	0	7	1	2	19	3	1	0	1	0.158
	High school or college	10	0	3	0	0	4	2	0	1	0	0.754
Occupation												0.178
	Employed	24	0	6	3	0	7	5	1	1	1	
	Unemployed	55	1	14	3	2	25	3	4	3	0	
Drug use method												0.402
	Injection drug user	51	1	13	4	2	19	6	1	4	1	
	Non-injection drug user	28	0	7	2	0	13	2	4	0	0	
Drug use duration												0.069
	<1 year	0	0	0	0	0	0	0	0	0	0	1.000
	1–2 years	12	0	4	0	0	6	2	0	0	0	0.735
	3–4 years	25	0	5	2	0	7	4	4	3	0	0.072
	5–10 years	23	1	3	4	0	12	2	0	0	1	0.060
	>10 years	19	0	8	0	2	7	0	1	1	0	0.117
HIV co-infection												0.061
	Yes	9	1	2	2	0	1	2	0	0	1	
	No	70	0	18	4	2	31	6	5	4	0	

HCV subtypes were determined by the sequence NS5B

^a^Rare subtypes including 1a, 2b, 6b, and 6u were removed from Fisher’s exact test. *p* < 0.05 were bolded

### Genotyping determination

Seventy-nine of those 116 RNA positive drug users were sent for sequencing for NS5B region of HCV, and nine subtypes were identified in total. Subtype 3a was the most common one, accounting for 40.5 % patients (Fig. 2), followed by subtype 1b, 3b, and 2a, which took 25.3, 10.1 and 7.6 %, respectively. Several rare subtypes were also identified, including five isolates identified as subtype 6a, four isolates as subtype 6b, two isolates as subtype 2b, one isolate as subtype 1a, and one isolate as subtype 6u. Compared to subtype 1b, 3a was more often found to be the younger and less often found among the married (Fisher’s exact test, *p* < 0.05), which indicates an emerging prevalence of subtype 3a infection among the high-risk population in Ningxia. In addition, subtype 1a, 2b, 6u and 6b were exclusively found in IDUs. Genotyping results based on Core region were present in Additional file 1: Supplementary Information 2 and Figure S1.

### Phylogenetic analysis

**Fig. 3:**
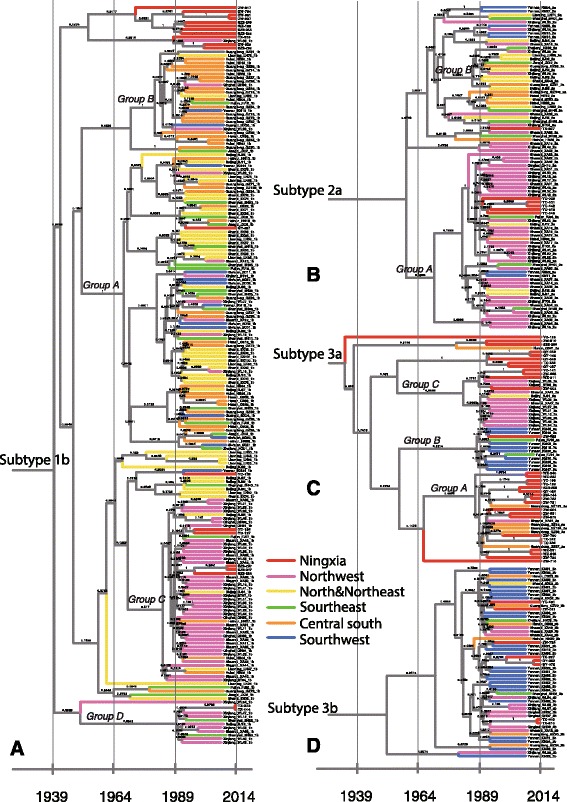
Bayesian phylogenetic tree based on NS5B from sequences from this study (labeled in red) along with 415 reference strains of 1b, 2a, 3a, and 3b. The geographic location of those sequences was mapped in Fig. 2. **a** The Bayesian tree of subtype 1b. **b** The Bayesian tree of subtype 2a. **c** The Bayesian tree of subtype 3a. **d** The Bayesian tree of subtype 3b

**Fig. 4:**
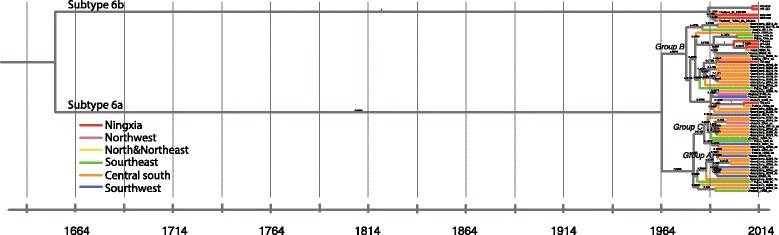
Bayesian phylogenetic tree based on NS5B from nine sequences (labeled in red) along with 55 references of 6a and 6b

Bayesian phylogenetic trees by subtypes were reconstructed for NS5B sequences to explore the possible transmission patterns of HCV infections, based on a large number of references from 17 provinces or municipalities and a few references of subtype 6u, 6b, 1a, and 2b strains from other provinces or countries outside China [19] (Fig. 1). For a better display, the Bayesian phylogenetic tree was split into five subtrees representing different subtypes (Figs. 3 and 4).

Subtype 1b used to be the most prevalent subtype in China, and strains from different regions were crossly distributed, suggesting simultaneous dissemination. In Fig. 3a, four groups, namely Group A to D, have been designated in previous studies with different geographic distributions features [19]. Most sequences from Ningxia, however, formed a cluster separately from Group A to D, indicating the local spreading of HCV.

Two major groups of 2a were reported: Group A contained sequences mainly from the northwest China, and Group B from various regions in China [19] (Fig. 3b). Five isolates, all collected from one rehabilitation center in Yinchuan, Ningxia, formed a cluster with the posterior probability of 0.99 in Group A. This cluster might descend from a common ancestor in northwest China dated back to year 1990s. In addition, two closely related isolates from Shizuishan, Ningxia were identified as subtype 2b. As subtype 2b was rarely reported in China, such isolates might imply possible real-world transmission pairs.

Subtype 3a sequences formed three separated geographic groups (Fig. 3c). Group A contained the majority of 3a isolates in Ningxia and reference sequences from central south China. Group B mainly contained sequences form southwest China. The rest isolates formed Group C, including sequences from neighboring Xinjiang province, in northwest China, which was in consistency to the known epidemic in the local area. Therefore, we hypothesized that subtype 3a was introduced into Ningxia in 1990s simultaneously from three regions mentioned above. Subtype 3b, unlike 3a, showed substantial mixture of originates, and sequences in Ningxia also contributed to the geographic interspersion (Fig. 3d).

The phylogeny of genotype 6 was reconstructed on the basis of nine Ningxia sequences and 55 reference sequences (Fig. 4). Three groups were noted among subtype 6a. We found that isolates of Ningxia were geographically interspersed among central south provinces in China. Therefore, the possible migration trend might arise from central south, spread to southeast and southwest, and then transmit to the northwest China sporadically. In addition, one close set of Ningxia sequences of subtype 6a was found with full posterior probability of 1.00 in Group B, implying a possible transmission network behind. Meanwhile, four Ningxia isolates clustered together were closely related to two 6b strains, D84262 and D37855, reported in Thailand [25].

The homology of every cluster of subtype was as follows: 1b 95.14 %, 2a 96.91 %, 2b 92.90 %, 3a 95.69 %, 3b 96.72 %, 6a 98.27 %, 6b 98.30 %. Highest homology was associated with subtype 6a and 6b, which suggested the late introduction of these subtypes to Ningxia; and the lowest homology (89.68 %) was associated with subtype 2b, indicating its long endemic history [26].

### Mutation analysis

We scanned for previously reported DAA-resistant mutations on NS5B, including S282T and C316N/Y in 79 NS5B sequences [27, 28]. No S282T samples were found, and seven C316N resistance samples were found in 1b subtype sequences, suggesting the natural occurrence of DAA-resistant mutations in this treatment-naïve population.

## Discussion

We found that seroprevalence of HCV was 0.34 % among individuals without drug use history and 15.8 % for drug users in Ningxia, based on a screening on individuals from Ningxia HIV/AIDS Sentinel Surveillance System. Ningxia was conventionally considered as a low-risk area, with the seroprevalence about 0.10 % among general population [1, 29]. Specialists and clinicians suggested a higher prevalence since some high-risk populations were not covered in the previous nationwide survey. Therefore, we mainly focused on drug users recruited from HIV surveillance system. We found that the prevalence among drug users was comparable to that of neighboring provinces but lower than the reported prevalence nationwide, that was, 66.97 % for IDUs and 18.97 % for non-injection drug users [8, 30].

Previous genotyping studies provided evidences to the hypothesis of two epidemics of HCV in China. Blood transfusion contributed to the first epidemic in China [31]. Subtype 1b, followed by 2a, was reported to be the most predominant subtypes in those studies that screened more blood donors [19, 32, 33]. Mandatory screening of blood and blood products was implemented in the 1990s to prevent the spreading of HCV [5]. Together with the increasing number of drug users, several studies have noticed a shift to the second epidemic: from subtype 1b to genotype 3 and 6 [34–37]. In addition, IDU is suspected as a factor that drives the emerging of new subtypes mainly due to genetic drift [38]. In our study, nine subtypes were identified, in which 3a (41.38 %) and 1b (25.29 %) were the predominant subtypes. Subtype 1a, 2b, 6u and 6b were not reported in Lu et al. [19], which was the largest genotyping study in China so far. The finding of multiple genotypes in Ningxia including fairly rare subtypes reflected the substantial mixture of HCV patients and accelerated propagation of the disease by modern transportation, internal migration, blood transfusion and IDU.

Studies on drug trafficking routes suggested the correlation of HIV transmission network and the drug smuggling routes from two major heroin-producing regions, “The Golden Triangle” (Myanmar, Laos and Thailand) and “The Golden Crescent” (Afghanistan and Pakistan) [39]. HCV shared the same way of transmission and thus the same epidemic features with HIV. The geographic features of HCV prevalence reinforced its spreading through these two drug trafficking routes [8]. Ningxia is located at the crossroad of the two drug trafficking routes geographically (Fig. 1). Phylogenetic analysis of subtype 3a also suggested the virus in Ningxia comes from both originates, Yunnan (bordering Myanmar and Laos) and Xinjiang (bordering Afghanistan and Pakistan) (Fig. 3c).

We found 11 co-infected patients of both HIV and HCV (13.9 %), which is much higher than the prevalence of 1.88 % among drug users in prior studies [8, 40]. This inconsistency may be due to the sampling bias, as samples collected in this study are from HIV/AIDS Sentinel Surveillance System. Besides, very few studies have been performed on mixed infection of different subtypes of HCV, which may be a hint for viral reconstruction, and a boost to fasten viral evolution [34]. Conventional sequencing methods based on short regions would not enable us to confirm hybrid genetics even if it exists. Further studies need to be performed on the detection of the intra-host viral population with deep sequencing [41].

With regard to the naturally occurring drug-resistance mutations, several single nucleotide polymorphisms (SNPs) of NS5B protease resistance have been previously reported in treatment-naïve hepatitis C patients [28, 42]. As the first DAA will be introduced to China soon, searching for drug-resistance SNPs may impact the planning for HCV treatment and predict the disease burden in the future. S282T was the most common one conferred resistance to NS5 nucleoside analogs inhibitors, such as Sofosbuvir, in subtype 1a, 1b and modest resistance in subtype 2a [43, 44]. In this study, we found no such resistance-bearing viral strains. However, C316N were detected in seven isolates among 1b sequences, which was reported to be associated with resistance to non-nucleotide inhibitor of NS5 protease [45–47]. Seven C316N-bearing sequences form two fairly close-related clusters, implying a direct transmission network behind, which calls for behavior study to confirm.

The findings of this study may impact the HCV controlling approaches in terms of screening, treatment, and patients management. First, several studies have suggested that interventions targeting drug users were effective approaches for HCV control [48, 49]. Continued screening, monitoring and treatment for drug users may reduce HCV incidence and prevalence, and could be highly cost-effectiveness. Second, needle exchange programs (NEP) and methadone maintenance treatment (MMT) may also be a feasible approach in controlling HCV transmission among IDUs. There are currently eight NEP/MMT sites operating in Ningxia, but still 23.1 to 44.4 % IDUs reported sharing needles in a recent survey conducted in 2015 [50, 51]. Our findings highlighted the necessity of the expansion of NEP in Ningxia and increased financial investment to such programs. In addition, treatment with the implementation of new DAAs could be highly effective, whereas their cost and value had been widely debated as the DAA treatment is much too expensive. Besides, the complex treatment regimen for HCV is further complicated by HIV co-infection [52, 53].

This study has several limitations. First, all serum samples were collected from individuals presented in HIV/AIDS Sentinel Surveillance System in 2013, and therefore the prevalence could not reflect that of general population. Further studies on the phylogeny on sequences from general population may present with a different pattern. Second, only NS5B and Core sequences were analyzed in our study. Whole genome sequencing performed on deep sequencing would provide more information on genotype distribution and mixed infection of various strains within one host [41]. Third, we did not collect enough clinical data, such as liver biopsy, aminotransferase/platelet ratio index (APRI) or FIB-4 scores, and thus were not able to stratify our patients by their liver status [54–56].

## Conclusion

In conclusion, the overall seroprevalence of HCV was 0.34 % among people with no history of drug use in Ningxia, while it was 15.8 % among drug users and 47.25 % among IDUs, based on a screening on individuals from Ningxia HIV/AIDS Sentinel Surveillance System. Nine subtypes, 1a, 1b, 2a, 2b, 3a, 3b, 6a, 6b and 6u were found among 79 isolates from drug users. 3a (41.38 %) followed by 1b (25.29 %) were the most predominant. Phylogenetic analysis suggested two possible originates of HCV transmission, accompanying the drug trafficking route in China. Our study suggested that drug use, especial IDU, is the most important risk factor for HCV infection, and management of drug users in Ningxia may play a crucial role in controlling the ongoing HCV epidemic.

## Additional file

Additional file 1: Supplementary information 1. Section 1: Introduction to HIV sentinel surveillance system. Section 2: Inclusion criteria of screened population. Supplementary information 2. Section 1: Sequence of NS5B and Core. Section 2: Measurement of agreement. Table S1: Comparing HCV genotyping results between the Core and NS5B regions. Supplementary information 3. Table S2: NS5B and Core region-specific primers. Supplementary information 4: Figure S1. The circular phylogenetic tree based on the Core sequences. (DOCX 545 kb)

## Abbreviations

APRI: Aminotransferase/platelet ratio index
CDC: Chinese Center of Disease Control and Prevention
DAA: Direct anti-viral agents
ELISA: Enzyme-linked immunosorbant assay
FSW: Female sex workers
HCC: Hepatocellular carcinoma
HCV: Hepatitis C virus
IDUs: Injection drug users
KAPs: key affecting populations
ML: Maximum-likelihood
MMT: Methadone maintenance treatment
MOH: Ministry of Health
MSM: Men who have sex with men
NEP: Needle exchange programs
SNPs: Single nucleotide polymorphisms
STD: Sexual transmitted disease
