# Supplementary material for: Molecular epidemiology of hepatitis C infections in Ningxia, China: genotype, phylogeny and mutation analysis
Source: Virol J. 2016 Oct 18;13:172. doi: 10.1186/s12985-016-0635-y (PMC5070218; doi:10.1186/s12985-016-0635-y)
Supplement: Additional file 1: — Supplementary information 1. Section 1: Introduction to HIV sentinel surveillance system. Section 2: Inclusion criteria of screened population. Supplementary information 2. Section 1: Sequence of NS5B and Core. Section 2: Measurement of agreement. Table S1: Comparing HCV genotyping results between the Core and NS5B regions. Supplementary information 3. Table S2: NS5B and Core region-specific primers. Supplementary information 4: Figure S1. The circular phylogenetic tree based on the Core sequences. (DOCX 545 kb) [file 12985_2016_635_MOESM1_ESM.docx]

Supplementary information for “**Molecular Epidemiology of Hepatitis C Infections in Ningxia, Northwest China: Genotype, Phylogeny and Mutation Analysis”**

**Supplementary information 1**

Section 1 Introduction to HIV sentinel surveillance system

Section 2 Inclusion criteria of screened population

**Supplementary information 2**

Section 1 Sequence of NS5B and Core

Section 2 Inclusion criteria of screened population

Table S1 Comparing HCV genotyping results between the Core and NS5B regions

**Supplementary information 3**

Table S2. NS5B and Core region-specific primers.

**Supplementary information 4**

Figure S1. The circular phylogenetic tree based on the Core sequences.

**Supplementary information 1**

**Section 1 Introduction to HIV sentinel surveillance system**

The HIV sentinel surveillance system was established in Jan 1995 under the support of the Ministry of Health (MOH), aiming at managing HIV/AIDS at a national level (Ministry of Health of China [MOH], National Center for AIDS Prevention and Control [NCAIDS] and Collaboration Group for National HIV Sentinel Surveillance Program., 1996). Nine kinds of key affecting populations were recruited and tested for HIV, syphilis, and HCV (National Center for AIDS/STD Control and Prevention. Chinese CDC., 2009).

**Section 2 Inclusion criteria of screened population**

1. Drug users: Individuals who used drugs, such as heroin, cocaine, opium, opiate analgesics, marijuana, and other so-called “nightclub drugs” including methamphetamine, ketamine, methylene dioxymetham-phetamine (MDMA), and Magu pills (mixed methamphetamine and caffeine). In addition, those drug users who have never injected drug were defined as “non-injection drug users (non-IDU).
2. Blood donors: Individuals who voluntarily donated blood from June to December 2013, in opposite to paid blood donors. Such voluntary blood donors with written informed consent were eligible.
3. Female sex workers (FSW): Females who provided commercial vaginal sex services for male clients currently or formerly.
4. Men who have sex with men (MSM): Males, aged 18 or older, who were engaged in oral or anal sex with other men during the last 12 months.
5. Sexual transmitted disease (STD) outpatients: Male outpatients who presented at selected STD clinics in Ningxia.
6. Long-distance truck drivers: Male truck drivers who engaged in long-distance transportation, staying overnight away from home at least three times during the most recent past three months.
7. Migratory populations: Individuals who have worked in provinces different from their registered residence during the past 3 years. Cases of travelling, schooling, seeking medical treatment, and serving in the army were excluded.
8. Pregnant women: Pregnant females who were scheduled for antenatal care in certain hospitals from June to December 2013.
9. Young students: Students registered in local colleges and technical schools from June to December 2013.

**Supplementary information 2**

**Section 1 Sequence of NS5B and Core**

NS5B and Core regions are both commonly sequenced for HCV genotyping. Several studies have suggested that NS5B is the most reliable region in HCV phylogenetic analysis (Cantaloube *et al.*, 2006). Therefore, we sequenced both regions, and mainly used NS5B for genotyping.

**Section 2 Measurement of agreement**

| **Core** | **NS5B** | | | | | | | | | | | | | | | | | |
| --- | --- | --- | --- | --- | --- | --- | --- | --- | --- | --- | --- | --- | --- | --- | --- | --- | --- | --- |
|  | *1a* | *1b* | | *2a* | | *2b* | | *3a* | | *3b* | | *6a* | | *6b* | | *6u* | | *Total* |
| 1a | 0 |  | |  | |  | |  | |  | |  | |  | |  | | 0 |
| 1b |  | 15 | |  | |  | | 3 | |  | | 1 | |  | |  | | 19 |
| 2a |  |  | | 0 | | 2 | |  | |  | |  | |  | |  | | 2 |
| 2b |  |  | |  | | 0 | |  | |  | |  | |  | |  | | 0 |
| 3a |  | 1 | |  | |  | | 16 | |  | |  | |  | |  | | 17 |
| 3b |  |  | |  | |  | | 5 | | 5 | |  | |  | |  | | 10 |
| 6a |  | 1 | |  | |  | |  | |  | | 2 | | 4 | |  | | 7 |
| 6b |  |  | |  | |  | |  | |  | |  | | 0 | |  | | 0 |
| 6u |  |  | |  | |  | |  | |  | | 1 | |  | | 0 | | 2 |
| Unclassified | 1 | 2 | | 6 | |  | | 8 | | 3 | | 1 | |  | | 1 | | 22 |
| Total | 1 | 20 | | 6 | | 2 | | 32 | | 8 | | 5 | | 4 | | 1 | | 79 |
| **Consistency rate** |  | |  | |  | |  | |  | |  | |  | |  | |  | 68.42% |
| **Kappa test** | Kappa-value = 0.598 (95% CI 0.437 - 0.758)  p-value < 0.05 | | | | | | | | | | | | | | | | |  |

We tested the agreement of two genotyping methods based on NS5B and Core by Kappa values (Landis & Koch, 1977). A p-value below 0.05 was considered as statistically significant. The extent of agreement is judged as follows: if Kappa is less than 0, “no agreement”, if 0-0.2, “slight agreement”, if 0.2-0.4, “fair agreement”, if 0.4-0.6, “moderate agreement”, if 0.6-0.8, “substantial agreement”, and if 0.8-1.0, “almost perfect agreement”. In most cases, two methods with a Kappa value over 0.6 are concluded as in consistency.

**Table S1. Comparing HCV genotyping results between the Core and NS5B regions.**

**Supplementary information 3**

**Table S2. NS5B and Core region-specific primers.**

| ***Region*** | ***Primer*** | ***Sequence*** | ***Loci**** | ***Size*** | ***Notes*** |
| --- | --- | --- | --- | --- | --- |
| NS5B | NS5B R1 | CTACCCCTACRGSRAGYAGGAGTAGGC | 9325-9351 | 1038 bp | External reverse |
|  | NS5B R2 | GRGCMYGRGACACGCTGTGATASATGTC | 9276-9303 |  | Internal reverse |
|  | NS5B F1 | GGSTTYTCGTATGAYACCMGBTGYTTTGA | 8247-8275 |  | External forward |
|  | NS5B F2 | GCTGYTTTGAYTCAACNGTCA | 8266-8286 |  | Internal forward |
| Core | Core R1 | CCARTTCATCATCATCATRTCCCASGCCAT | 1293-1322 | 1245 bp | External reverse |
|  | Core R2 | GGRTGDCCNGKRTARATNGARCARTTRCA | 1251-1279 |  | Internal reverse |
|  | Core F1 | GGCGACACTCCACCATGAATCACT | 18-41 |  | External forward |
|  | Core F2 | AATCACTCCCCTGTGAGGAACTACTGT | 35-61 |  | Internal forward |

* Loci number was referred to HCV genome NC_004102.

**Supplementary information 4**

Fig S1. The circular phylogenetic tree based on the Core sequences from 59 subjects that were positive in Core sequencing. Different subtypes were shown in different colors. The pie chart inside showed percentages of different HCV subtypes, indicated in the same color coding used of the circular tree.


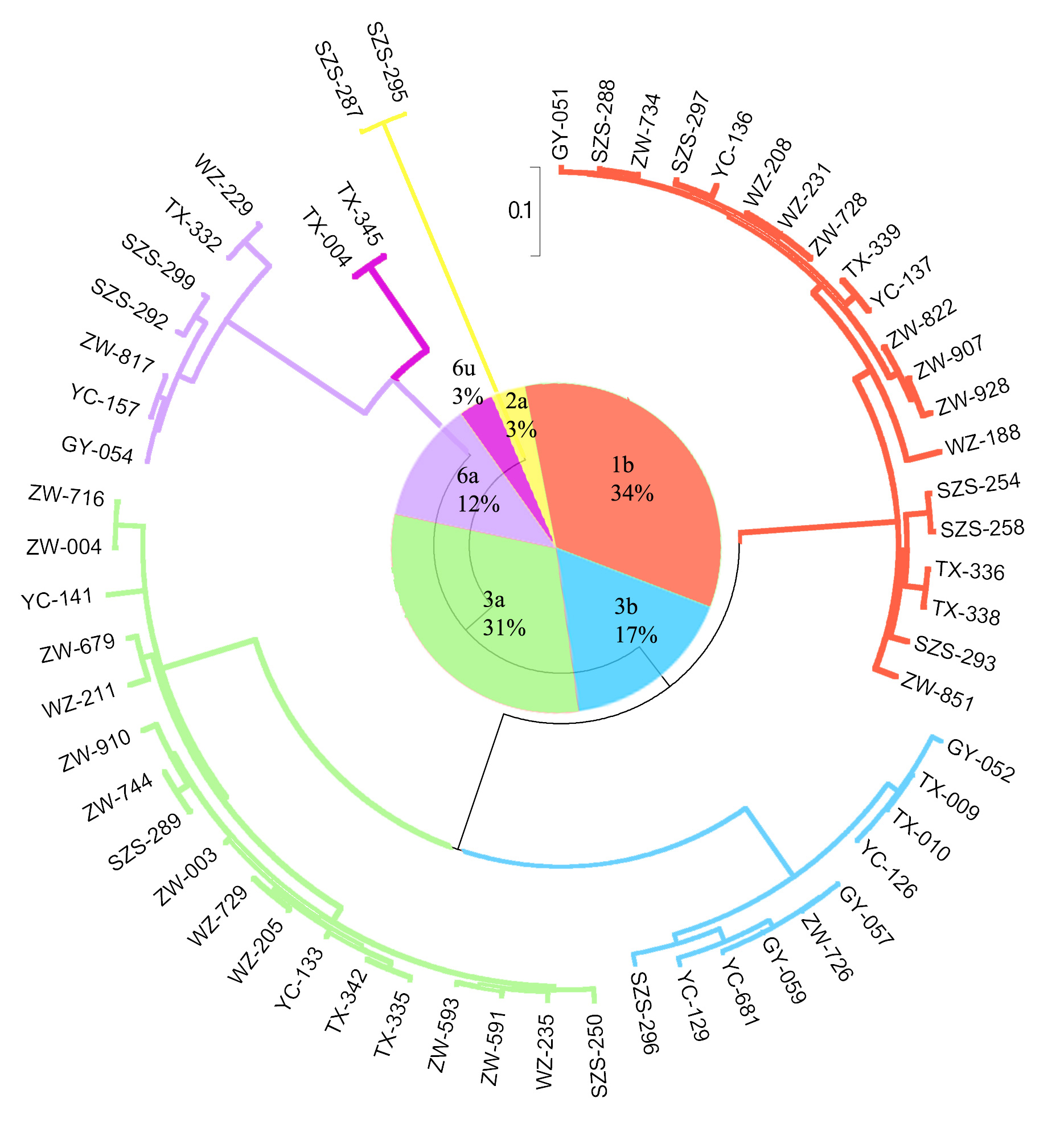


**Cantaloube, J. F., Laperche, S., Gallian, P., Bouchardeau, F., de Lamballerie, X. & de Micco, P. (2006).** Analysis of the 5' noncoding region versus the NS5b region in genotyping hepatitis C virus isolates from blood donors in France. *J Clin Microbiol* **44**, 2051-2056.

**Landis, J. R. & Koch, G. G. (1977).** The measurement of observer agreement for categorical data. *Biometrics* **33**, 159-174.

**Ministry of Health of China (MOH). National Center for AIDS Prevention and Control (NCAIDS) and Collaboration Group for National HIV Sentinel Surveillance Program. (1996).** Set up of national sentinel surveillance of HIV infection in China and its report in 1995. . *Zhongguo Xing Bing Ai Zi Bing Fang Zhi* **2**, 193–197.

**National Center for AIDS/STD Control and Prevention. Chinese CDC. (2009).** Guideline for HIV&STD Sentinel Surveillance in China. In *Beijing: National Center for AIDS/STD Control and Prevention, Chinese Center for Disease Control and Prevention*.
